# Genome-Wide Characterization of Citrus NBS-LRR Genes and Integrative Analysis of a Candidate Gene Associated with Alternaria Brown Spot-Related QTL

**DOI:** 10.3390/plants15081191

**Published:** 2026-04-13

**Authors:** Yilu Li, Chengnan Kang, Ru Zhang, Boping Wu, Kai Xu, Jiajie Chen, Meiyan Wang, Jinhua Liu, Haijie Ma

**Affiliations:** 1 Key Laboratory of Quality and Safety Control for Subtropical Fruit and Vegetable, Ministry of Agriculture and Rural Affairs, Collaborative Innovation Center for Efficient and Green Production of Agriculture in Mountainous Areas of Zhejiang Province, College of Horticulture Science, Zhejiang A&F University, Hangzhou 311300, China; 13600670457@163.com (Y.L.); 13386658617@163.com (C.K.); zhang_ru0507@163.com (R.Z.); bopingwu@zafu.edu.cn (B.W.); xukai@zafu.edu.cn (K.X.); wangmeiyan0606@163.com (M.W.); 2 Yangsheng TANG Co., Ltd., No. 181, Geyazhuang, Xihu District, Hangzhou 310024, China; jjchen28@mail.yst.com.cn

**Keywords:** citrus, NBS-LRR gene family, Alternaria brown spot, QTL-associated candidate gene, genome-wide analysis

## Abstract

Alternaria brown spot, caused by the tangerine pathotype of *Alternaria alternata*, is a destructive fungal disease affecting citrus production worldwide. Nucleotide-binding site-leucine-rich repeat (NBS-LRR) genes constitute a major class of plant immune receptors; however, their genome-wide characteristics and potential association with Alternaria brown spot resistance loci in citrus remain poorly understood. In this study, we performed a comprehensive genome-wide identification and comparative analysis of NBS-LRR genes across representative citrus species. A total of 417 and 326 NBS-LRR genes were identified in *Citrus reticulata* and *Citrus clementina*, respectively, and were classified into NL, CNL, TNL, and RNL subfamilies based on domain architecture. Phylogenetic reconstruction, gene structure analysis, conserved motif composition, chromosomal distribution, synteny relationships, and promoter cis-element profiling collectively revealed considerable structural variation and lineage-specific expansion of the NBS-LRR gene family in citrus genomes. By integrating previously reported quantitative trait locus (QTL) data for Alternaria brown spot, we identified several NBS-LRR genes located within a resistance-associated genomic interval on chromosome 3. Among these, a candidate gene, designated LRR2, exhibited differential transcriptional responses upon pathogen inoculation and displayed distinct sequence variations between citrus genotypes. Structural modeling and molecular docking analyses suggested potential binding interfaces between LRR2 and multiple host-selective toxins, although the biological relevance of these interactions requires further experimental validation. Subcellular localization assays in *Nicotiana benthamiana* showed that LRR2 is distributed in both the nucleus and cytoplasm. Notably, transient overexpression of LRR2 triggered hypersensitive response-like cell death and H_2_O_2_ accumulation. Collectively, this study provides a comprehensive overview of the citrus NBS-LRR gene family and presents a multifaceted characterization of a QTL-anchored candidate gene. These findings establish a genomic and molecular framework for further functional investigations of citrus–*Alternaria* interactions.

## 1. Introduction

Citrus is one of the most economically important fruit crops worldwide and represents the largest group of fresh fruit production globally. Species within the genus *Citrus* (Rutaceae), including mandarins, oranges, grapefruits, pummelos, and related hybrids, are widely cultivated across tropical and subtropical regions. Despite the remarkable expansion of citrus cultivation, productivity and fruit quality are continually threatened by diverse biotic stresses, among which fungal diseases constitute a major limiting factor for sustainable industry development. Alternaria brown spot (ABS), caused by *Alternaria alternata* tangerine pathotype, is one of the most destructive fungal diseases affecting citrus production worldwide [1]. The disease primarily infects young leaves, shoots, and fruits, leading to necrotic lesions, defoliation, fruit drop, and twig dieback [1]. In severe cases, infected fruits become unsuitable for fresh market sales, resulting in substantial economic losses. Under favorable environmental conditions characterized by warm temperatures, high humidity, and frequent rainfall, the disease can spread rapidly and become difficult to control [2]. Current management strategies largely depend on repeated fungicide applications, typically ranging from four to ten sprays per growing season and even more in highly susceptible orchards [3]. However, excessive reliance on chemical control not only increases production costs but also poses environmental risks and may promote the development of fungicide-resistant pathogen populations [4]. Therefore, developing alternative disease management strategies based on host genetic improvement and molecular understanding of host–pathogen interactions is of considerable importance.

The pathogenicity of the *A. alternata* tangerine pathotype is closely associated with the production of host-selective toxins (HSTs), especially ACT toxins [5,6], which are low-molecular-weight polyketide compounds and key virulence determinants. Two major isoforms (ACT-I and ACT-II) have been identified with ACT-I showing significantly stronger phytotoxic activity and inducing typical necrotic lesions on susceptible citrus tissues at extremely low concentrations [5,7]. ACT toxin biosynthesis is mediated by a specific gene cluster in the fungal genome [8]. Genetic disruption or silencing of toxin biosynthetic genes abrogates toxin production and pathogenicity, with no significant effects on fungal vegetative growth and sporulation, demonstrating that ACT toxins are specifically required for fungal virulence but not normal fungal development [6]. HST production is not unique to the *A. alternata* tangerine pathotype; at least six other Alternaria pathotypes produce structurally related HSTs, including AF toxin (strawberry pathotype), AK toxin (Japanese pear pathotype), AT toxin (tobacco pathotype), AM toxin (apple pathotype), and ACR toxin (rough lemon pathotype) [5,8]. Interestingly, although these pathotypes share high genomic similarity and certain toxins exhibit conserved structural backbones, they display strict host specificity. This phenomenon indicates that toxin perception and downstream signaling in host cells are controlled by highly specific molecular determinants [5,9]. Ultrastructural observations have revealed distinct cellular targets for different toxins: ACT, AF, and AK toxins primarily disrupt plasma membrane integrity, causing membrane depolarization and electrolyte leakage; ACR and AT toxins affect mitochondrial function; and AM toxin targets chloroplasts. Despite extensive cytological characterization, the molecular mechanisms underlying toxin recognition in citrus remain largely unknown [5,10,11]. Evidence from toxin analog studies further supports the hypothesis that host cells may contain specific toxin-interacting components. For example, AF-I and AF-II toxins share high structural similarity; however, only AF-I exhibits toxicity toward strawberry cells, whereas AF-II is non-toxic. Pre-treatment with AF-II can competitively reduce AF-I toxicity, suggesting the presence of specific receptor-mediated interactions [12]. Whether similar molecular recognition mechanisms exist in citrus for ACT toxin perception remains an open question. Recent advances have begun to elucidate the molecular basis of effector biology in *A. alternata*. For instance, studies have identified key transcription factors and biosynthetic genes governing host-selective toxin production and have revealed the presence of conditionally dispensable chromosomes that carry essential virulence determinants [6,7,8]. Moreover, emerging evidence suggests that fungal effectors may target host immune components to suppress defense responses, although the specific targets in citrus remain largely unknown. Further characterization of these effector mechanisms will be critical for understanding host–pathogen interactions and developing durable resistance strategies. Genetic studies have demonstrated that resistance to ABS in certain citrus genotypes follows a Mendelian inheritance pattern [13,14]. Clementine mandarin exhibits stable and complete resistance to ABS, and genetic mapping has localized a resistance-associated quantitative trait locus (QTL) to a 366 kb region on chromosome 3 [15,16]. This region contains 24 annotated genes, including multiple predicted resistance gene candidates. However, due to the complexity of citrus genomes and the technical challenges associated with genetic transformation, functional validation of candidate genes within this interval remains limited.

Plant disease resistance is commonly mediated by nucleotide-binding site-leucine-rich repeat (NBS-LRR, also referred to as NLR) proteins, which constitute a major class of intracellular immune receptors [17]. Canonical NBS-LRR proteins contain a central NB-ARC domain responsible for nucleotide binding and hydrolysis, a C-terminal LRR domain implicated in specificity determination and protein–protein interactions, and variable N-terminal domains such as Toll/interleukin-1 receptor (TIR), coiled-coil (CC), or RPW8 motifs. Based on domain architecture, NBS-LRR genes are classified into TNL, CNL, RNL, and NL subfamilies [18,19]. Upon recognition of pathogen-derived effectors or effector-induced modifications of host proteins, NLR proteins undergo conformational rearrangements that activate downstream immune signaling cascades, frequently culminating in hypersensitive response (HR)-associated programmed cell death [20,21,22]. Numerous NBS-LRR genes have been functionally characterized in model and crop plants. For example, *RPS4/RRS1* in *Arabidopsis recognizes* the bacterial effector PopP2 and activates the EDS1-dependent signaling pathway [23]; *Pm21* confers resistance to powdery mildew in wheat [24], and *XA21* in rice represents a paradigm for receptor-mediated immune signaling coordination [25]. These examples underscore the central role of NBS-LRR genes in plant immunity and highlight their evolutionary diversification in response to pathogen pressure. The number of NBS-LRR genes identified in citrus genomes can vary substantially across studies, mainly due to differences in species sampling, gene identification pipelines, and criteria for retaining partial or incomplete NLR structures. In the present study, we focused on canonical, full-length NBS-LRR genes containing both NB-ARC and LRR domains to ensure high-confidence candidates for functional analysis, while a recent study using a more inclusive R-gene pipeline recovered a larger number of putative NLR loci [26]. Despite these recent efforts, a comprehensive genome-wide analysis of the NBS-LRR gene family across representative citrus species remains insufficiently explored. Furthermore, the distribution and evolutionary patterns of NBS-LRR genes with respect to ABS-associated QTL intervals have not been systematically investigated. Given the enrichment of predicted resistance genes within the chromosome 3 QTL region, a global characterization of citrus NBS-LRR genes may provide key insights for candidate gene prioritization and future functional studies.

In this study, we performed a genome-wide identification and comparative analysis of NBS-LRR genes across representative citrus species. We characterized their phylogenetic relationships, gene structure organization, conserved motif composition, chromosomal distribution, syntenic relationships, and promoter cis-regulatory elements. Furthermore, by integrating previously reported ABS-associated QTL data, we identified NBS-LRR genes located within the chromosome 3 interval and performed multifaceted molecular characterization of a selected candidate gene. This work provides a comprehensive genomic framework for subsequent studies of citrus-*Alternaria* interactions and provides foundational resources for understanding NBS-LRR gene family evolution in citrus.

## 2. Results

### 2.1. Alternaria Brown Spot Sensitivity Analysis of C. reticulata and C. clementina

**Figure 1:**
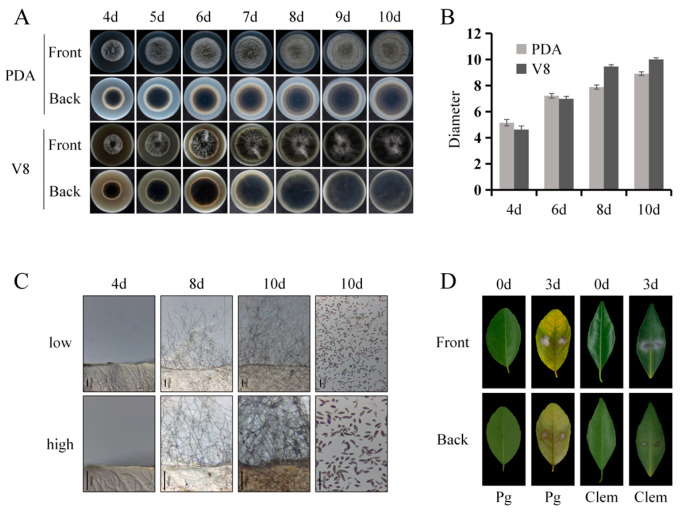
Morphological characteristics of *A. alternata* and pathogenicity assays on citrus leaves. (**A**) Colony morphology of *A. alternata* cultured on PDA and V8 media at 26 °C in darkness. (**B**) Colony diameter measurements of *A. alternata* grown on PDA and V8 media. Data represent the mean ± SD. (**C**) Microscopic observation of aerial hyphae and conidia of *A. alternata*. Conidia are multicellular, septate, and morphologically diverse. The scale bars in the figures represent 100 μm. (**D**) Pathogenicity assay showing mycelial plug inoculation on leaves of Dancy mandarin (*C. reticulata*) and Clementine mandarin (*C. clementina*).

To evaluate differences in susceptibility to Alternaria brown spot, the fungal isolate *A. alternata* ZJU1 was first cultured and morphologically characterized. When grown on potato dextrose agar (PDA) at 26 °C in darkness, colonies reached an average diameter of 5.15 cm after 4 days, with radial growth continuing thereafter (Figure 1A,B). On PDA, colonies initially appeared gray on the surface and gradually darkened with extended incubation; the reverse side displayed a white peripheral margin and a predominantly brown central region (Figure 1A). On V8 medium under the identical incubation conditions (26 °C, dark), colony diameter reached 4.63 cm after 4 days and increased progressively thereafter. While early growth on V8 was slightly slower than on PDA, the growth rate became marginally higher at later stages (Figure 1A,B). Colonies grown on V8 displayed a brown surface, and the reverse side was largely brown in the central region (Figure 1A). Microscopic examination revealed brown aerial hyphae on V8 medium. Conidiation initiated approximately 6 days post-incubation, and mature conidia were observed by day 10 (Figure 1C). Conidia harvested from V8 plates using sterile water were multicellular, septate, and morphologically diverse, exhibiting obclavate, ellipsoid, or ovoid shapes with brown pigmentation (Figure 1C). Pathogenicity assays were performed by mycelial plugs of *A. alternata* inoculated onto leaves of *C. reticulata* and *C. clementina*. At 72 h post-inoculation, *C. reticulata* leaves developed distinct brown necrotic lesions on both adaxial and abaxial surfaces. Lesions expanded radially along leaf veins, displaying typical symptoms of Alternaria brown spot (Figure 1D). In contrast, although fungal mycelia continued to grow on the inoculation plug, no visible lesions were observed at the inoculation sites on *C. clementina* leaves within the same time frame (Figure 1D).

### 2.2. Genome-Wide Identification and Phylogenetic Characterization of NBS-LRR Genes in C. reticulata and C. clementina

**Figure 2:**
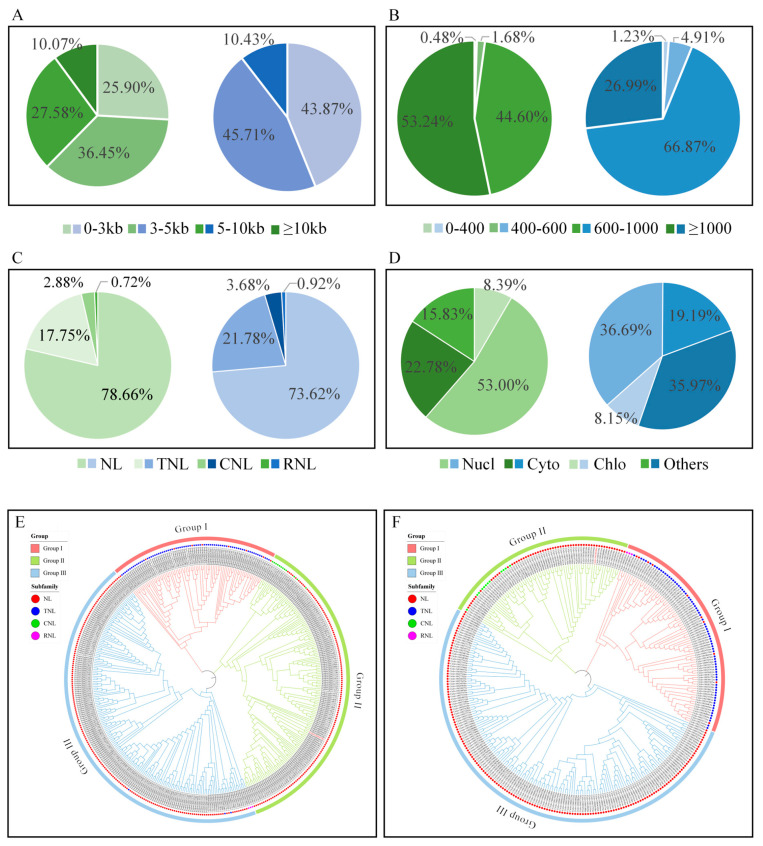
Phylogenetic and structural analyses of NBS-LRR proteins in *C. reticulata* and *C. clementina*. (**A**) Pie chart showing gene length distribution of NBS-LRR family members in *C. reticulata* and *C. clementina*. Left: Green represents *C. reticulata*; Right: Blue represents *C. clementina*. (**B**) Pie chart showing amino acid length distribution of NBS-LRR proteins in *C. reticulata* and *C. clementina*. Left: Green represents *C. reticulata*; Right: Blue represents *C. clementina*. (**C**) Proportional distribution of NBS-LRR subfamilies (NL, CNL, TNL, and RNL) in *C. reticulata* and *C. clementina*. Left: Green represents *C. reticulata*; Right: Blue represents *C. clementina*. (**D**) Predicted subcellular localization distribution of NBS-LRR proteins in *C. reticulata* and *C. clementina*. Left: Green represents *C. reticulata*; Right: Blue represents *C. clementina*. (**E**) Phylogenetic tree constructed from NBS-LRR protein sequences of *C. reticulata*. (**F**) Phylogenetic tree constructed from NBS-LRR protein sequences of *C. clementina*.

A genome-wide survey of NBS-LRR genes was performed across five representative *Citrus* species (Ponkan mandarin, Clementine mandarin, pummelo, sweet orange, and kumquat) and one closely related species (*Poncirus trifoliata*). The number of NBS-LRR genes varied substantially among species (Table 1). Specifically, 417 and 326 NBS-LRR-encoding genes were identified in the genomes of *C. reticulata* and *C. clementina*, respectively (Tables S1 and S2). The identified NBS-LRR genes exhibited considerable structural diversity. Gene lengths ranged from approximately 700 bp to 400 kb, with exon–intron numbers varying from 0 to 13 and cDNA lengths ranged from 400 bp to 10,000 bp (Figure 2A). The predicted proteins showed substantial size variation, with amino acid lengths ranging from 200 to 3000 residues and estimated molecular weights of 15–350 kDa (Figure 2B). These features reflect extensive heterogeneity in gene architecture and coding capacity within the NBS-LRR family. Based on the domain composition preceding the NB-ARC region, NBS-LRR proteins were classified into four subfamilies: NL (NBS-LRR lacking a defined N-terminal domain), CNL (CC-NBS-LRR), TNL (TIR-NBS-LRR), and RNL (RPW8-NBS-LRR). In *C. reticulata*, 328 NL, 12 CNL, 74 TNL, and 3 RNL genes were identified (Figure 2C). In *C. clementina*, 240 NL, 12 CNL, 71 TNL, and 3 RNL genes were detected. The NL subfamily represented the largest proportion in both species, accounting for 78.66% and 73.62% of total NBS-LRR genes in *C. reticulata* and *C. clementina*, respectively. TNL genes formed the second largest subgroup (17.75% and 21.78%), while CNL and RNL genes represented relatively minor fractions (2.88% and 3.68% for CNL; 0.72% and 0.92% for RNL) (Figure 2C). The differential proportions of subfamilies suggest lineage-specific expansion patterns within citrus genomes. Subcellular localization prediction revealed that the majority of NBS-LRR proteins were predicted to localize to the nucleus and cytoplasm. In *C. reticulata*, 52.99% of proteins were predicted to localize to the nucleus, 22.78% to the cytoplasm, 8.39% to chloroplasts, and 6.23% to both the nucleus and cytoplasm. In *C. clementina*, 36.69% were predicted to localize to the nucleus, 19.18% to the cytoplasm, 8.15% to chloroplasts, and 4.31% to both the nucleus and cytoplasm (Figure 2D). To examine evolutionary relationships among NBS-LRR proteins, multiple sequence alignments were performed separately for each species, followed by phylogenetic tree construction. The resulting phylogenies revealed three major clades: Group I contained predominantly NL and TNL members; Group II comprised NL and CNL members; and Group III consisted mainly of NL genes with a smaller number of RNL, CNL, and TNL members. NL genes were distributed across all three clades, while TNL genes were primarily clustered within Group I and CNL genes were mainly grouped in Group II, indicating conserved clustering patterns associated with domain architecture. Notably, RNL genes were located in Group III in *C. reticulata*, whereas in *C. clementina* they were positioned within Group I, suggesting potential divergence in evolutionary trajectories between the two species (Figure 2E,F).

### 2.3. Conserved Domain Architecture and Motif Composition of Citrus NLR Proteins

**Figure 3:**
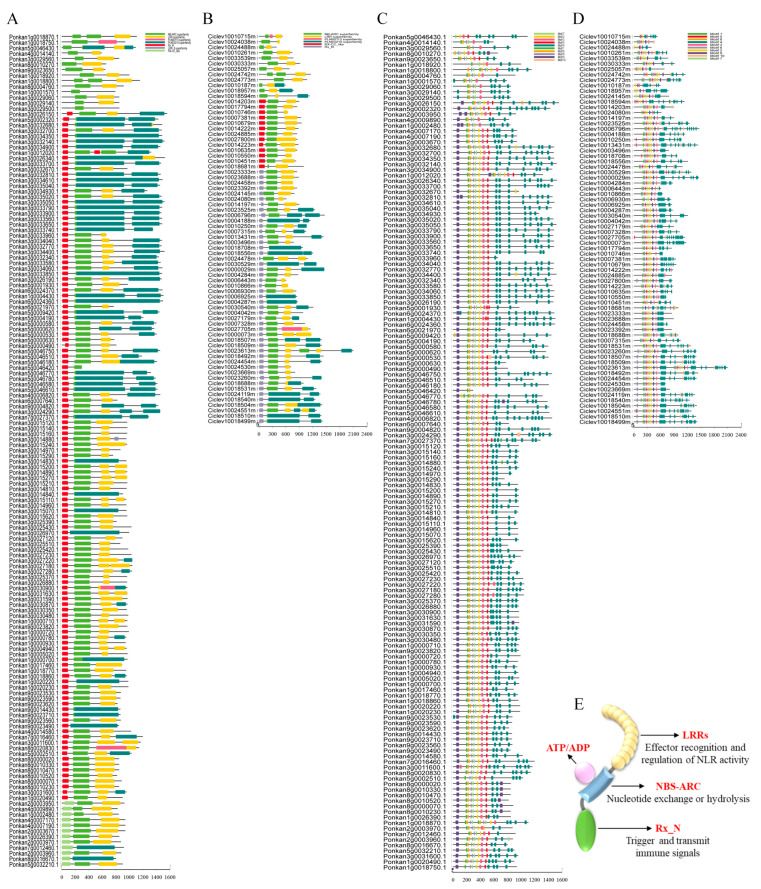
Conserved domain architecture and motif composition of NLR proteins in *C. reticulata* and *C. clementina*. (**A**) Visualization of conserved domain organization of NLR proteins in *C. reticulata*. (**B**) Conserved motif distribution of NLR proteins in *C. reticulata* as identified by MEME analysis. (**C**) Visualization of conserved domain organization of NLR proteins in *C. clementina*. (**D**) Conserved motif distribution of NLR proteins in *C. clementina*. (**E**) Schematic representation of the canonical modular structure of NLR proteins.

To gain insight into the structural organization and potential functional characteristics of citrus NLR proteins, conserved domain architecture and motif composition were systematically analyzed and visualized. Domain annotation using the NCBI Conserved Domain Database confirmed that all identified candidate proteins harbored the characteristic NB-ARC domain and C-terminal leucine-rich repeat (LRR) domain, which are hallmarks of canonical NLR proteins and form the structural framework for protein–protein interactions. In addition to these core domains, a subset of proteins possessed N-terminal TIR, CC, or RPW8 domains, resulting in distinct structural classes, including TNL, CNL, and RNL subfamilies. The specific domain combinations align with their established classification and are consistent with their potential involvement in distinct immune signaling pathways, such as pattern-triggered immunity (PTI) and effector-triggered immunity (ETI) (Figure 3A,B). NLR proteins are modular receptors, with each domain fulfilling distinct functional roles. The central NB-ARC domain functions as a nucleotide-binding molecular switch that regulates receptor activation through ATP binding and hydrolysis. The C-terminal LRR domain is generally implicated in effector recognition or specificity determination, while the N-terminal domains (TIR, CC, or RPW8) are associated with downstream signal transduction and plant immune response activation. Collectively, these domains form a coordinated system for pathogen perception and signal relay. To further evaluate structural conservation within the citrus NLR family, conserved motifs were predicted using the MEME suite. A total of 10 conserved motifs with high sequence similarity were identified across all NLR proteins. Members of the same phylogenetic subgroup exhibited highly consistent motif composition, number, and sequential order, indicating structural conservation within evolutionary lineages (Figure 3C,D). These conserved motif patterns may contribute to subgroup-specific interaction interfaces or regulatory properties. Integration of domain architecture and motif distribution allowed the construction of a representative schematic model illustrating the modular organization of citrus NLR proteins (Figure 3E). The structural diversity observed across subfamilies suggests potential functional differentiation within the citrus NLR repertoire. In particular, proteins containing the TIR-NBS-LRR configuration correspond to the canonical TNL architecture commonly associated with intracellular immune receptors, consistent with their putative role in effector-responsive signaling pathways.

### 2.4. Cis-Regulatory Element Profiles of Citrus NLR Gene Promoters

**Figure 4:**
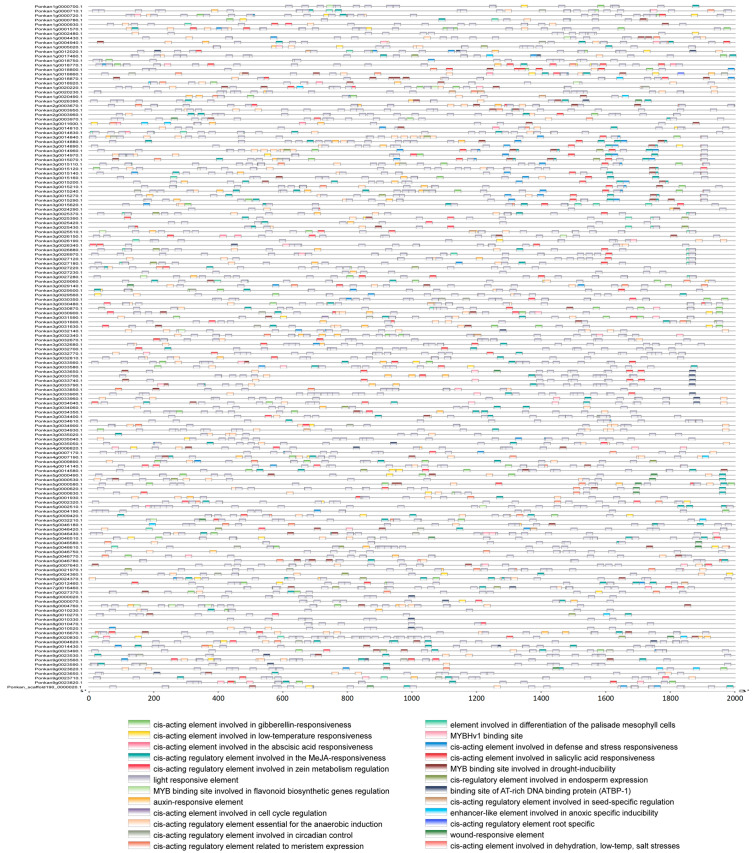
Distribution of cis-regulatory elements in the promoter regions of NLR genes in *C. reticulata*.

Gene expression is largely influenced by cis-acting regulatory elements located within promoter regions. To assess the regulatory potential of citrus NLR genes, the 2000 bp sequences upstream of the transcription start site were analyzed for cis-regulatory elements. The promoter regions were enriched with diverse functional motifs associated with stress responses, hormone signaling, and developmental regulation. Notably, multiple defense-related elements were identified, including salicylic acid-responsive elements (TCA elements), methyl jasmonate (MeJA)-responsive elements, and abscisic acid (ABA)-responsive elements. Cis-regulatory elements identified in *C. reticulata* and *C. clementina* were categorized into five functional groups: abiotic stress-responsive elements, cell cycle-related elements, plant growth and development-associated elements, hormone-responsive elements, and transcription-related elements. A substantial proportion of NBS-LRR genes contained hormone-responsive cis-elements. In *C. reticulata* and *C. clementina*, 302 and 226 genes, respectively, contained ABA-responsive elements; 292 and 210 genes contained MeJA-responsive elements; 189 and 138 genes harbored salicylic acid-responsive TCA-elements; 178 and 134 genes contained auxin-responsive TGA-elements; and 212 and 175 genes possessed gibberellin-responsive elements. Stress-related cis-elements were also widely distributed among NBS-LRR genes. Among genes containing complete NBS-LRR domains, all 417 genes in *C. reticulata* and 324 genes in *C. clementina* contained light-responsive elements. Additionally, 152 and 146 genes were associated with defense- and stress-responsive elements; 72 and 47 genes contained circadian regulation-related elements; 322 and 251 genes harbored anaerobic induction elements; 227 and 190 genes contained drought-inducible MYB binding sites; and 163 and 141 genes included low-temperature-responsive elements in *C. reticulata* and *C. clementina*, respectively. Considerable variation in both the number and diversity of cis-elements was observed among individual genes. For example, Ponkan1g0009410.1 contained 43 cis-regulatory elements, whereas Ciclev10027643m.1 contained only 1. Similarly, Ponkan3g0016450.1 harbored 12 distinct types of cis-elements, while Ciclev10027643m.1 contained only a single element type. These differences indicate substantial heterogeneity in promoter architecture among citrus NLR genes (Figure 4).

### 2.5. Chromosomal Distribution and Synteny Analysis of Citrus NLR Genes

**Figure 5:**
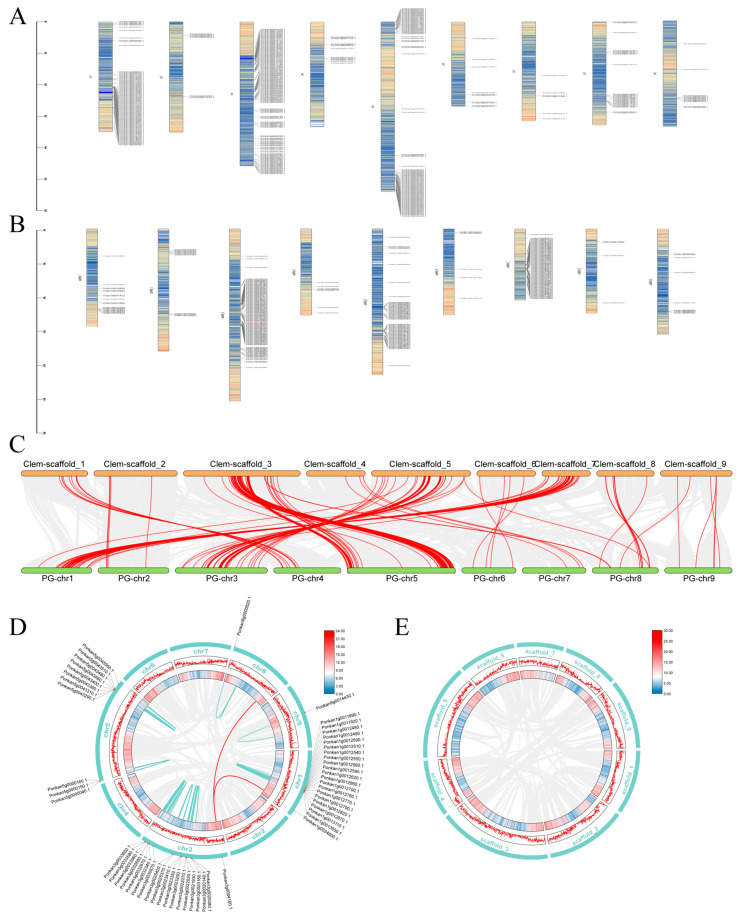
Chromosomal distribution, QTL localization, and synteny analysis of NLR genes in *C. reticulata* and *C. clementina*. (**A**) Chromosomal distribution of NBS-LRR genes in *C. reticulata*. (**B**) Chromosomal distribution of NBS-LRR genes in *C. clementina*. (**C**) Interspecific synteny analysis of NBS-LRR genes between *C. reticulata* and *C. clementina*. (**D**) Intraspecific synteny analysis of NBS-LRR genes in *C. reticulata*. (**E**) Intraspecific synteny analysis of NBS-LRR genes in *C. clementina*.

Chromosomal mapping revealed that NBS-LRR genes were distributed across all nine chromosomes in both *C. reticulata* and *C. clementina*, with a small number of genes located on unanchored scaffolds (five in *C. reticulata* and twelve in *C. clementina*). The chromosomal distribution of NBS-LRR genes was markedly uneven in both species. In *C. reticulata*, 97 genes were located on chromosome 1, 8 on chromosome 2, 141 on chromosome 3, 13 on chromosome 4, 98 on chromosome 5, 10 on chromosome 6, 6 on chromosome 7, 27 on chromosome 8, and 12 on chromosome 9 (Figure 5A). In *C. clementina*, 20 genes were mapped to chromosome 1, 12 to chromosome 2, 109 to chromosome 3, 7 to chromosome 4, 65 to chromosome 5, 6 to chromosome 6, 81 to chromosome 7, 4 to chromosome 8, and 10 to chromosome 9 (Figure 5B). In *C. reticulata*, the majority of NBS-LRR genes were concentrated on chromosomes 1, 3, and 5, accounting for 23.26%, 33.81%, and 23.50% of the total, respectively. In *C. clementina*, genes were predominantly distributed on chromosomes 3, 5, and 7, representing 33.43%, 19.93%, and 24.85% of the total NBS-LRR complement. Visualization of chromosomal localization indicated that NLR genes were unevenly distributed and tended to form clustered regions in certain chromosomal segments (Figure 5A,B). Such clustering patterns are consistent with tandem duplication events and are commonly observed in rapidly evolving resistance gene families. Interspecific collinearity analysis identified multiple homologous gene pairs derived from segmental duplication events in the genomes of *C. reticulata* and *C. clementina* (Figure 5C). Intraspecific synteny analysis identified multiple homologous gene pairs derived from segmental duplication events within the *C. reticulata* genome. A total of 89 homologous gene pairs were detected among Ponkan NBS-LRR genes, including two interchromosomal pairs, while the remaining duplicated pairs were located on the same chromosome (Figure 5D). In contrast, no homologous gene pairs were detected among NBS-LRR genes in *C. clementina* under the applied criteria (Figure 5E). The lower number of duplicated gene pairs detected in *C. clementina* may also be explained by genome assembly characteristics: the *C. clementina* genome was generated using Sanger sequencing, and some duplicated sequences may have been collapsed or excluded as repetitive regions during assembly [26], making it less comparable to recent long-read-based genome assemblies. Combined chromosomal distribution and synteny analyses provide insights into the evolutionary dynamics of the citrus NLR gene family. The observed patterns suggest that tandem and segmental duplication events have contributed to family expansion to varying degrees in different citrus lineages. The distinct genomic position and duplication pattern of NBS-LRR2 further highlight its unique evolutionary status within the family (Figure 5D,E). Detailed chromosomal localization and physical coordinates of all NBS-LRR genes are provided in Tables S1 and S2.

### 2.6. LRR2 Is Located Within an Alternaria Brown Spot-Associated QTL and Exhibits Distinct Predicted Binding Patterns with Host-Selective Toxins

**Figure 6:**
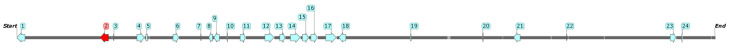
Gene analysis within an Alternaria brown spot-associated QTL interval in citrus. Genomic distribution and transcriptional orientation of the 24 annotated genes within the 366 kb QTL region on chromosome 3.

A previous genetic study by Cuenca et al. mapped an Alternaria brown spot (ABS) resistance locus in *C. clementina* through hybridization between resistant and susceptible cultivars, followed by phenotypic evaluation, genome sequencing, and linkage map construction [15,16]. The resistance-associated locus was delimited to a 366 kb interval on chromosome 3 of the Clementine genome (Figure 6).

**Figure 7:**
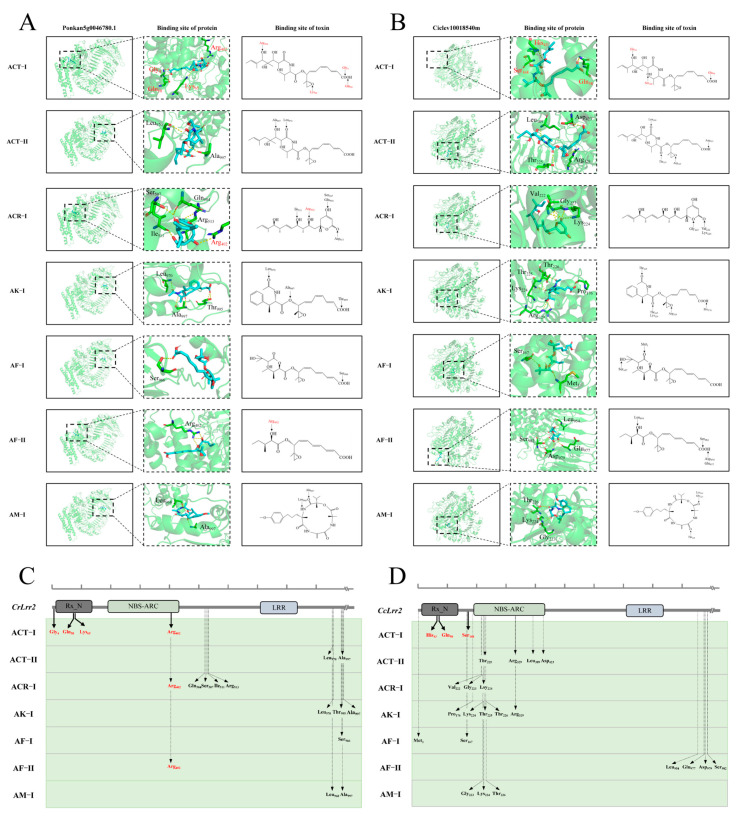
Molecular docking analysis of NBS-LRR2 proteins from *C. reticulata* and *C. clementina* with ACT-I and six additional host-selective toxins. (**A**) Visualization of predicted docking complexes between Ponkan NBS-LRR2 and seven toxins (ACT-I, ACT-II, ACR-I, AK-I, AF-I, AF-II, and AM-I). (**B**) Visualization of predicted docking complexes between Clementine NBS-LRR2 and the seven toxins. (**C**) Distribution of predicted toxin-binding sites along the Ponkan NBS-LRR2 protein structure. (**D**) Distribution of predicted toxin-binding sites along the Clementine NBS-LRR2 protein structure.

Annotation of this genomic region identified 24 predicted genes, including 6 genes encoding leucine-rich repeat (LRR)-containing proteins, 5 of which belong to the canonical NBS-LRR family. Transcriptome analysis of resistant and susceptible citrus genotypes after *A. alternata* inoculation identified four NBS-LRR genes within the QTL region with differential expression patterns (Ciclev10023260, Ciclev10018540, Ciclev10018510, and Ciclev10018897) (Table 2) [27]. Based on its expression pattern and genomic location, Ciclev10018540 was selected for further functional analysis and hereafter named LRR2. To investigate potential structural interactions between LRR2 and HSTs, three-dimensional (3D) protein models of LRR2 from *C. reticulata* and *C. clementina* were constructed and used for molecular docking with seven small-molecule toxins: ACT-I, ACT-II, ACR-I, AK-I, AF-I, AF-II, and AM-I (Table 3). Structural comparison showed that all tested toxins except AM-I share a conserved polyketide backbone with a 9,10-epoxy-8-hydroxy-9-methyl core structure, while ACT-I differs from the other six toxins in side-chain configuration. In *C. reticulata*, molecular docking predicted potential binding between LRR2 and all seven HSTs; ACT-I was predicted to interact with residues near the N-terminal and within the NB-ARC domain (Gly4, Gln58, Lys65, and Arg402). Other toxins exhibited predicted binding sites distributed across different regions of the protein, primarily within or downstream of the NB-ARC domain. Notably, except for ACR-I and AF-II, which shared Arg402 as a predicted binding residue with ACT-I, the remaining toxins did not overlap with ACT-I in predicted binding positions. ACT-I binding sites were concentrated near the N-terminal Rx_N and NB-ARC regions, whereas most other toxins were predicted to interact with regions located downstream of the NBS core domain (Figure 7A,C). In *C. clementina*, LRR2 also exhibited predicted interactions with all seven toxins, although the binding patterns differed from those observed in *C. reticulata*. ACT-I was predicted to bind near the N-terminal region, including residues His43, Gln58, and Ser168. In contrast to Ponkan, no other toxin shared predicted binding residues with ACT-I. Other toxins were predicted to interact predominantly within the NB-ARC domain or adjacent non-conserved regions. Importantly, none of the non-ACT toxins showed predicted binding within the Rx_N domain in Clementine (Figure 7B,D). Collectively, molecular docking analysis revealed species-specific differences in the predicted toxin-binding interfaces of LRR2. The distinct binding patterns, particularly those associated with ACT-I, suggest potential structural divergence between LRR2 homologs in resistant and susceptible citrus genotypes, although experimental validation (e.g., in vitro binding assays, surface plasmon resonance) is required to determine the biological relevance of these predicted interactions.

### 2.7. Subcellular Localization and Function Analysis of LRR2

**Figure 8:**
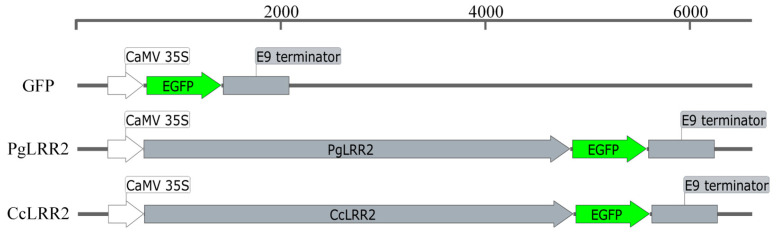
Schematic representation of the *LRR2* overexpression constructs. EGFP, enhanced green fluorescent protein coding sequence; CaMV 35S, constitutive cauliflower mosaic virus 35S promoter; E9 terminator, transcriptional terminator sequence; *PgLRR2*, *LRR2* coding sequence derived from the Alternaria brown spot-susceptible cultivar Ponkan (*C. reticulata*); *CcLRR2*, *LRR2* coding sequence derived from the Alternaria brown spot-resistant cultivar Clementine (*C. clementina*).

To determine the subcellular localization of LRR2, the full-length coding sequences of *LRR2* from *C. reticulata* and *C. clementina* were cloned downstream of the CaMV 35S promoter. The stop codon was removed to generate in-frame C-terminal GFP (green fluorescent protein) fusion constructs (LRR2-GFP). The recombinant binary vectors were introduced into *A. tumefaciens* strain GV3101 and transiently expressed in *N. benthamiana* leaves via agroinfiltration. An empty GFP vector lacking the *LRR2* coding sequence served as the control (Figure 8).

**Figure 9:**
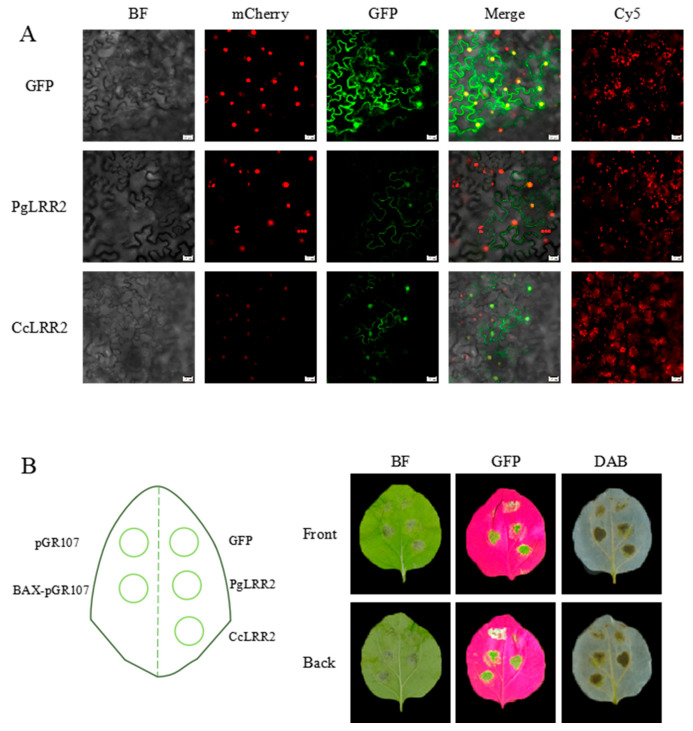
Subcellula localization and function analysis of LRR2. (**A**) Subcellular localization analysis of PgLRR2 and CcLRR2 in *N. benthamiana*. BF: bright-field micrographs of the infiltrated *N. benthamiana* leaf regions; mCherry: fluorescence micrographs showing nuclear-localized RFP (excitation wavelength optimized for RFP) in *N. benthamiana* cells; GFP (upper): fluorescence micrographs showing GFP signal (excitation wavelength optimized for GFP) in *N. benthamiana* cells; Merge: merged images of bright-field, RFP, and GFP channels; Cy5: chlorophyll autofluorescence detection channel; Left GFP: control group infiltrated with *Agrobacterium* harboring empty GFP vector; PgLRR2: *Agrobacterium*-infiltrated group overexpressing the *C. reticulata* LRR2-GFP fusion construct; CcLRR2: *Agrobacterium*-infiltrated group overexpressing the *C. clementina* LRR2-GFP fusion construct. (Scale bar: 20 μm). (**B**) Hypersensitive response (HR) and H_2_O_2_ accumulation induced by transient overexpression of LRR2 in *N. benthamiana* leaves. pGR107 (empty vector) served as a negative control; BAX-pGR107 (pro-apoptotic BAX protein) served as a positive control for HR induction; GFP (empty GFP vector) served as a vector control for the LRR2-GFP fusion constructs; PgLRR2 and CcLRR2 represent constructs overexpressing the LRR2 gene from *C. reticulata* and *C. clementina*, respectively, fused with GFP. A schematic diagram of a tobacco leaf (left) illustrates the five infiltration sites corresponding to each construct. Representative images (right) are arranged in two rows (adaxial and abaxial surfaces) and three columns: BF (bright-field) showing leaf appearance under white light; GFP (fluorescence) showing images captured under a GFP excitation light source; DAB showing leaves stained with DAB and subsequently destained with ethanol to visualize hydrogen peroxide accumulation as brown precipitates.

At 48 h post-infiltration, green fluorescence was detected in both control and LRR2-GFP-expressing leaves. The control GFP signal appeared relatively strong and uniformly distributed. Confocal laser scanning microscopy of abaxial epidermal cells showed that GFP fluorescence in the control was present in both the cytoplasm and nucleus. Co-localization with a nuclear marker (mCherry-tagged nuclear localization signal) confirmed nuclear accumulation of the GFP signal. In both Ponkan and Clementine constructs, LRR2-GFP fluorescence was observed in the cytoplasm and nucleus, indicating a dual subcellular distribution pattern. This localization pattern was consistent with prior in silico predictions (Figure 9A). Notably, transient overexpression of LRR2 in tobacco cells was associated with altered fluorescence patterns compared to the GFP control. In LRR2-expressing cells, fluorescence boundaries appeared diffuse, signal intensity was reduced, and in some cells GFP fluorescence was barely detectable. These changes were not observed in the control group. To further investigate whether these phenotypic alterations were associated with immune activation, we assessed hypersensitive response (HR) and reactive oxygen species (ROS) accumulation following transient overexpression of LRR2 (Figure 9B). Compared with the negative controls (pGR107 and GFP), infiltration of BAX-pGR107 (positive control) induced typical HR-related tissue collapse. Similarly, leaves expressing PgLRR2 and CcLRR2 exhibited visible necrotic patches at the infiltration sites under bright-field observation. Consistent with this, 3,3′-diaminobenzidine (DAB) staining revealed brown precipitates indicating hydrogen peroxide (H_2_O_2_) accumulation in LRR2-expressing leaves, whereas no such staining was observed in the negative controls. These results indicate that transient overexpression of LRR2 triggers HR-like cell death and ROS production in *N. benthamiana*.

## 3. Discussion

Alternaria brown spot, caused by the *A. alternata* tangerine pathotype, represents a classic example of HST-mediated plant disease, wherein the pathogen’s production of ACT toxins determines virulence toward susceptible citrus genotypes [28,29]. Despite extensive cytological characterization of toxin-induced cellular disruption, the molecular basis of toxin perception and immune activation in citrus has remained elusive. In this study, we integrated genome-wide characterization of the citrus NBS-LRR gene family with QTL-targeted candidate gene analysis, providing a genomic framework for understanding citrus–*Alternaria* interactions and a detailed characterization of a candidate gene potentially involved in ABS response.

Genome-wide analysis identified 417 and 326 NBS-LRR genes in *C. reticulata* and *C. clementina*, respectively, revealing substantial variation in family size between closely related species. This observation aligns with the rapidly evolving nature of plant NLR gene families, which undergo birth-and-death evolution in response to local pathogen pressure [30]. The family sizes documented here are comparable to those reported in other woody perennials, including grapevine and apple [31,32]. Such interspecific variation reflects lineage-specific evolutionary trajectories shaped by distinct pathogen pressures and genome duplication histories [33,34]. The predominance of the NL subclass in both citrus species represents a striking feature of the citrus NLR repertoire. While TNL and CNL subclasses have been extensively characterized in model plants [35,36,37], the functional significance of NL-type genes remains poorly understood. Variation in NLR subfamily composition, including differential retention of NL-type genes, has been documented across rosid species, reflecting lineage-specific evolutionary trajectories [38].

Chromosomal distribution analysis revealed uneven clustering of NLR genes across both citrus genomes, with significant enrichment on chromosomes 1, 3, and 5 in *C. reticulata* and chromosomes 3, 5, and 7 in *C. clementina*. Such clustering patterns are commonly associated with tandem duplication events, which drive NLR diversification through sequence exchange and unequal crossing over [39,40,41,42]. This clustering tendency mirrors findings in numerous plant species, including *Arabidopsis*, rice, and poplar [43,44,45]. Intraspecific synteny analysis identified 89 duplicated gene pairs in *C. reticulata* but few in *C. clementina*, indicating differential evolutionary trajectories that may reflect distinct selection pressures or domestication history [46,47]. Conserved domain and motif analyses demonstrated that citrus NLR proteins maintain the canonical NB-ARC and LRR architecture. The NB-ARC domain functions as a molecular switch regulating receptor activation, while the LRR domain is implicated in recognition specificity [48,49]. Conservation of core motifs within phylogenetic subclades indicates functional constraints on nucleotide-binding mechanisms, while LRR diversification contributes to recognition specificity evolution [50]. This structural modularity provides a plausible basis for pathogen perception [51]. Promoter cis-element profiling revealed enrichment of hormone-responsive and stress-associated motifs, including elements responsive to salicylic acid, methyl jasmonate, and abscisic acid. The widespread presence of SA- and MeJA-related elements is noteworthy, as these hormones coordinate defense against biotrophic and necrotrophic pathogens, respectively [52,53]. Although ABS displays necrotrophic features, the integration of multiple hormone-responsive elements suggests that citrus NLR genes participate in complex immune crosstalk [54,55]. The presence of light-responsive and abiotic stress-related elements further suggests integration of stress signals with immune regulation [56,57].

By integrating previously reported ABS-associated QTL data, a resistance locus was mapped to a 366 kb genomic region on chromosome 3 [16,58], where 24 annotated genes were identified, including five NBS-LRR genes. Transcriptome analysis revealed four NLR genes displaying differential expression following pathogen inoculation, among which Ciclev10018540 (designated *LRR2*) was prioritized. Localization within a resistance-associated QTL strengthens its candidacy, consistent with the established role of NLR genes in disease resistance [38,40,59]. However, co-localization does not establish causality, and resistance may involve coordinated action of multiple genes [60]. Molecular docking predicted potential binding interfaces between LRR2 and ACT-I plus six other structurally related HSTs, with distinct predicted binding residues between *C. reticulata* and *C. clementina*, suggesting structural divergence between LRR2 homologs in resistant and susceptible genotypes. ACT-I binding sites in Ponkan were concentrated near the N-terminal and NB-ARC regions, whereas in Clementine they were distributed differently. These species-specific docking patterns may reflect sequence polymorphisms that alter local structural conformation. Notably, all these docking results are purely computational predictions; technical constraints related to protein stability, host-selective toxin purification and assay setup currently prevent the performance of in vitro binding experiments to validate these interactions. The structural comparison among toxins revealed conserved backbones with side-chain variations, aligning with classical studies demonstrating competitive inhibition between structurally related toxins [61]. While docking simulations provide valuable structural hypotheses, they require experimental validation. Whether NLR proteins directly perceive HSTs remains uncertain, and biochemical approaches such as co-immunoprecipitation or surface plasmon resonance are needed.

Functional validation of candidate resistance genes remains a major challenge in citrus, largely due to the low efficiency of genetic transformation in this genus. In this context, alternative strategies such as forward genetic screening via mutagenesis, as demonstrated by Wang et al. (2025) using ARTP mutagenesis combined with whole-genome resequencing in bacteria, may offer valuable approaches for identifying functionally relevant mutations in plant immune receptors [62]. Applying such mutagenesis-based approaches to citrus or model systems could help elucidate the in vivo functions of NLR genes, including the candidate *LRR2* characterized in this study, by linking sequence variation to phenotypic changes in disease response [62]. While our transient overexpression assays in *N. benthamiana* have provided evidence for *LRR2*-mediated immune activation, we confirm that stable *in planta* validation of *LRR2* function remains an important priority for future work, and we intend to pursue this using improved citrus transformation systems or gene-editing protocols as they become available. These optimized genetic tools will enable direct validation of *LRR2*’s biological function in citrus, filling the current gap of in vivo genetic evidence for this candidate resistance gene.

Subcellular localization assays demonstrated that LRR2 is distributed in both the nucleus and cytoplasm, consistent with many NLR proteins that shuttle between subcellular compartments to coordinate immune signaling [63,64,65,66]. Nuclear accumulation is associated with transcriptional reprogramming, while cytoplasmic localization facilitates effector perception [67,68]. In this study, transient overexpression of LRR2 was experimentally confirmed to induce HR-like cell death and H_2_O_2_ accumulation in *N. benthamiana*. These immune phenotypes are fully validated by DAB staining and phenotypic observation. However, further quantitative measurement of ROS levels, expression analysis of cell-death marker genes, and characterization of downstream signaling pathways are still needed to elucidate the detailed molecular mechanisms underlying LRR2-mediated immune activation [69]. Understanding toxin–host interaction mechanisms has both theoretical and practical implications. From an evolutionary perspective, ABS represents a model for studying co-evolution between HSTs and plant immune receptors [70,71]. Identification of NLR genes associated with toxin perception may refine the classical gene-for-gene hypothesis. From an applied standpoint, structural insights could inform resistance breeding through marker-assisted selection or development of small-molecule inhibitors that disrupt toxin–host interactions.

We acknowledge that the present study focused on two representative citrus species (*C. reticulata* and *C. clementina*), which serve as well-characterized susceptible and resistant models for Alternaria brown spot. Expanding the analysis to a broader range of citrus species would provide additional insights into NLR family evolution and resistance mechanisms. Future studies incorporating more diverse germplasm will help to further validate and extend our findings.

In conclusion, this study provides a comprehensive overview of the citrus NBS-LRR gene family and characterizes a QTL-localized candidate gene potentially involved in ABS response. The findings reveal substantial variation in NLR repertoire size, predominance of NL-type genes, chromosomal clustering indicative of tandem duplication, conserved structural features, and promoter architectures enriched for hormone-responsive elements. The localization of *LRR2* within an ABS-associated QTL—together with its differential expression, predicted species-specific toxin-binding interfaces, and dual subcellular distribution—provides a basis for further investigation into its potential role in citrus resistance. Future studies integrating genome editing, biochemical interaction assays, and comprehensive phenotyping will be necessary to determine whether *LRR2* functionally contributes to toxin perception and immune activation.

## 4. Materials and Methods

### 4.1. Plant Materials and Growth Conditions

*Citrus clementina* and *Citrus reticulata* were used in this study. *C. clementina* plants were obtained from the Citrus Research Institute (Chongqing, China), and *C. reticulata* plants were provided by Prof. Hongye Li (Zhejiang University). All plants were cultivated at the experimental orchard of Zhejiang A&F University (Hangzhou, Zhejiang Province, China) under standard field management conditions. For transient expression assays, *Nicotiana benthamiana* plants were grown in a controlled growth chamber at 25 °C under a 16 h light/8 h dark photoperiod.

### 4.2. Fungal Strain and Culture Conditions

The *Alternaria alternata* tangerine pathotype strain (ZJU1) was kindly provided by Prof. Jiao Chen of Zhejiang University. The strain was maintained on potato dextrose agar (PDA) at 26 °C in darkness. For activation, mycelial plugs were transferred onto fresh PDA plates and incubated at 26 °C for 5–8 days. For conidiation, mycelial plugs were transferred from PDA to V8 medium and cultured at 26 °C in darkness for 9–12 days. Plates were unsealed on day 3 to allow conidia formation. Conidia were harvested by washing plates with sterile distilled water and filtered through lens paper. Spore concentration was adjusted to 1 × 10^6^ spores mL^−1^ using a hemocytometer. To ensure reproducibility, mycelial plugs were inoculated in triplicate on both PDA and V8 media, with daily diameter measurements taken using the cross-bracketing method and averaged from repeated assessments.

### 4.3. Pathogen Inoculation Assays

Mycelial Plug Inoculation: Fresh and healthy leaves of *C. clementina* and *C. reticulata* were placed on moist filter paper in sealed trays. Mycelial plugs (5 mm diameter) from the actively growing edge of fungal colonies were placed on the leaf surface (mycelium in contact with tissue). Inoculated leaves were incubated at 26 °C in darkness for 72 h. Conidial Suspension Inoculation: Conidial suspensions were adjusted to 1 × 10^5^ spores mL^−1^. Ten microliters of suspension was dropped onto the leaf surface. Leaves were incubated at 26 °C in darkness for 72 h under high humidity. For reproducibility, at least three healthy leaves from both *C. clementina* and *C. reticulata* were subjected to pathogen inoculation, with non-inoculated leaves from the same plants serving as controls. Disease progression was assessed by tracking the expansion of lesions on inoculated leaves over time, and symptom development was recorded using photography.

### 4.4. Genome-Wide Identification and Classification of NBS-LRR Genes in C. clementina and C. reticulata

To systematically identify NBS-LRR genes in the *C. clementina* and *C. reticulata* genomes, a hidden Markov model (HMM)-based screening strategy was used. The HMM profile of the conserved NB-ARC domain (Pfam accession: PF00931), a signature domain of NBS-LRR proteins, was downloaded from the Pfam database (http://pfam.xfam.org/). The downloaded HMM profile was used as a query to search against the complete protein sequence datasets of both citrus species using the “Simple HMM Search” module implemented in TBtools-II. An E-value cutoff of <1 × 10^−5^ was used to reduce false-positive hits and ensure the reliability of candidate sequence identification. All candidate proteins from the initial HMM search were further validated to confirm the presence and structural integrity of the NB-ARC domain. Domain verification was performed using the NCBI Conserved Domain Database (CDD) Batch Search tool (accessed on 12 October 2025) and InterProScan (version 108.0). Only sequences containing a complete and intact NB-ARC domain were retained for further analysis. Subsequently, proteins were screened for the presence of leucine-rich repeat (LRR) domains to ensure their classification within the NBS-LRR family. Redundant entries and sequences exhibiting severely truncated or incomplete conserved domains were manually inspected and excluded. The final non-redundant dataset of confirmed NBS-LRR genes was compiled for each species. Based on the architecture of their N-terminal domains, the identified NBS-LRR proteins were further categorized into subfamilies, including TNL (TIR-NBS-LRR), CNL (CC-NBS-LRR), RNL (RPW8-NBS-LRR), and NL (NBS-LRR lacking a recognizable N-terminal signaling domain), according to established domain-based classification criteria.

### 4.5. Gene Structure, Conserved Motif, Domain Architecture and Phylogenetic Analyses of Citrus NBS-LRR Proteins

To comprehensively characterize the structural features and evolutionary relationships of NBS-LRR genes in *C. clementina* and *C. reticulata*, multiple levels of sequence and structural analyses were conducted. Gene structure information, including exon–intron organization and positional distribution, was extracted from the corresponding genome annotation GFF3 files. The exon–intron structures of individual NBS-LRR genes were visualized using the “Visualize Gene Structure” module implemented in TBtools, enabling comparative analysis of gene architecture across subfamilies. Conserved motif analysis of NBS-LRR proteins was performed using the MEME suite (https://meme-suite.org/) to identify conserved sequence patterns within the protein family. Full-length amino acid sequences were submitted for analysis, with the maximum number of motifs set to 10 while other parameters were maintained at default settings. The identified motif information was subsequently imported into TBtools and visualized using the “Visualize Motif Pattern (from MEME suite)” function to examine motif composition and distribution among different NBS-LRR subgroups. To further investigate conserved domain architecture, candidate NBS-LRR protein sequences were analyzed using the NCBI Conserved Domain Database (CDD) via the Batch-CDD search tool. The resulting domain annotations were processed and graphically represented using the “Visualize Domain Pattern (from NCBI Batch-CDD)” module in TBtools, allowing comparative assessment of domain types, structural organization, and domain distribution patterns across identified proteins. For phylogenetic analysis, multiple sequence alignments of all identified NBS-LRR proteins were performed using MAFFT with default parameters. The aligned sequences were manually trimmed in MEGA12 to remove poorly aligned regions and gaps. Phylogenetic trees were constructed using the Neighbor-Joining (NJ) method with 1000 bootstrap replicates to assess branch support. The resulting trees were visualized and annotated using the Interactive Tree of Life (iTOL, https://itol.embl.de/) platform for graphical refinement and classification of subfamilies.

### 4.6. Chromosomal Localization and Synteny Analysis of NBS-LRR Genes in Citrus

To investigate the chromosomal distribution patterns of NBS-LRR genes in *C. clementina* and *C. reticulata*, gene location information was extracted from the corresponding genome annotation (GFF3) files. The physical positions of identified NBS-LRR genes were mapped onto individual chromosomes using TBtools. Gene density distribution across chromosomes was calculated using the “Gene Density Profile” module, generating heatmap files that reflect gene clustering tendencies. Chromosomal localization maps were subsequently visualized using the “Gene Location Visualize from GTF/GFF” function in TBtools by integrating genome annotation files, NBS-LRR gene ID lists, and gene density data. To explore the evolutionary relationships and duplication patterns of NBS-LRR genes, both interspecific and intraspecific synteny analyses were performed based on the MCScanX algorithm implemented in TBtools. For interspecific synteny analysis, genome sequence files and corresponding GFF annotations from citrus species were analyzed using the “One Step MCScanX” pipeline to identify collinear gene pairs. Collinearity files were generated and further processed to highlight NBS-LRR family members within conserved genomic blocks. Syntenic relationships were visualized to assess conserved gene organization between species. For intraspecific synteny analysis, chromosome scaffold information and gene position data were first generated from genome sequence files using TBtools utilities. MCScanX was subsequently employed to identify duplicated gene pairs within each genome. Collinearity files were processed and merged to generate chromosome-anchored syntenic gene pair datasets. Identified NBS-LRR gene pairs were manually curated and highlighted for visualization. Final graphical representations, including chromosome ideograms, gene density heatmaps, and syntenic linkages, were generated using the “Advanced Circos” module in TBtools. These analyses enabled the assessment of chromosomal distribution patterns, tandem and segmental duplication events, and evolutionary conservation of NBS-LRR genes in citrus genomes.

### 4.7. Promoter Cis-Regulatory Element Analysis of Citrus NBS-LRR Genes

To investigate potential regulatory features of NBS-LRR genes in *C. clementina* and *C. reticulata*, promoter sequences were extracted and analyzed for cis-regulatory elements. The 2000 bp genomic regions upstream of the transcription start site (TSS) of each identified NBS-LRR gene were retrieved from genome annotation files using the “GXF Sequences Extract” and “Fasta Extract (Recommended)” functions in TBtools. The extracted promoter sequences were subsequently submitted to PlantCARE for the prediction of putative cis-acting regulatory elements. The resulting annotation files were curated to remove redundant or non-informative elements, and identified motifs were classified according to their functional categories, including hormone-responsive elements, stress-responsive elements, development-related elements, and transcription-associated elements. The distribution and composition of cis-regulatory elements were then visualized using the “Basic BioSequence View” module in TBtools, enabling comparative analysis of promoter architecture between citrus species.

### 4.8. Transcriptome-Based Candidate Gene Screening

Transcriptome data were obtained from a previous study [27], including resistant *C. clementina* and susceptible *C. reticulata* leaves inoculated with wild-type and ACT toxin-deficient *A. alternata* strains. Samples were collected at 0 h and 48 h post inoculation with three independent biological replicates per treatment and genotype. Sequencing reads were aligned to the reference genome using HISAT2. Gene expression levels were quantified as FPKM/TPM, and differential expression analysis was performed using DESeq2. Genes with |log_2_FC| ≥ 1 and adjusted *p*-value (FDR) ≤ 0.05 were considered significantly differentially expressed. Candidate genes located within the 366 kb ABS resistance QTL interval on chromosome 3 were prioritized, and *LRR2* was selected for further analysis based on its stable differential expression pattern.

### 4.9. Molecular Docking Analysis of NBS-LRR2 Proteins and Small-Molecule Ligands

Three-dimensional (3D) structures of LRR2 from *C. reticulata* and *C. clementina* were predicted using PaddleHelix (HelixFold) (https://paddlehelix.baidu.com/), an AI-driven protein structure modeling platform [72]. Model quality was evaluated based on pTM, pLDDT and Ranking Score values (pTM > 0.5, pLDDT > 70 for reliable models). The pTM score ranges from 0 to 1, with a value greater than 0.5 indicating that the predicted structure is close to the true conformation and considered reliable. The pLDDT score ranges from 0 to 100, with values above 70 indicating high confidence and above 90 indicating very high confidence. The Ranking Score ranges from 0 to 1 and represents a comprehensive evaluation; higher values indicate greater reliability. The resulting models were exported in PDB format. The 3D structures of selected small-molecule ligands were obtained from the PubChem database. For compounds lacking available 3D conformations, 2D structures were downloaded and converted into 3D models using ChemBio3D 15.0 software, followed by energy minimization to optimize molecular geometry. The optimized ligand structures were saved in PDB format. Protein–ligand molecular docking was performed using the CB-Dock2 web server (http://183.56.231.194:8001/cb-dock2/index.php, accessed on 16 September 2025) in Auto Blind Docking mode, which integrates cavity detection, docking, and homologous template fitting based on AutoDock Vina (version 1.1.2) [73]. The prepared receptor and ligand PDB files were uploaded to the platform, and docking simulations were conducted under default parameters. Docking results were evaluated based on Vina scores (binding free energy estimates). Vina score lower than -6 indicate favorable binding affinity, suggesting good binding activity between the protein and the ligand. Lower scores correspond to tighter binding. The top-ranked docking conformations were downloaded and visualized using PyMOL (version 3.1.6.1). Protein–ligand complexes were rendered with customized color settings, and hydrogen bond interactions were identified using polar contact analysis. Key amino acid residues involved in ligand binding were annotated and highlighted to illustrate potential interaction interfaces. Final molecular interaction figures were generated for graphical presentation.

### 4.10. Subcellular Localization Analysis of NBS-LRR2

The full-length coding sequence (CDS) of the target gene, without the stop codon, was cloned in-frame into the pCAMBIA2300-GFP vector to generate a NBS-LRR2-GFP fusion construct under the control of the CaMV 35S promoter. The empty GFP vector was used as a control. Recombinant plasmids were introduced into *Agrobacterium tumefaciens* strain GV3101 using the freeze–thaw method. Single positive colonies were cultured in LB medium containing appropriate antibiotics at 28 °C with shaking at 200 rpm for 24–36 h until saturation. Bacterial cultures were then diluted 1:10 into fresh antibiotic-containing LB medium and grown to an OD_600_ of 0.6–0.8. Cells were harvested by centrifugation at 5000 rpm for 10 min at room temperature and resuspended in MES infiltration buffer. The final bacterial suspension was adjusted to OD_600_ values of 0.1, 0.4, or 0.8 and incubated at room temperature in the dark for 2–4 h prior to infiltration. Healthy and fully expanded leaves of *N. benthamiana* plants were selected for transient expression assays. *Agrobacterium* suspensions were infiltrated into the abaxial side of leaves using a needleless syringe, generating transiently water-soaked areas. Infiltrated plants were maintained at 25 °C in darkness for 24 h, followed by incubation under a 16 h light/8 h dark photoperiod at 25 °C for an additional 48 h. At 72 h post-infiltration, leaf discs from the infiltrated regions were excised and mounted on glass slides with sterile water. GFP fluorescence and chlorophyll autofluorescence were observed using a confocal laser scanning microscope to determine the subcellular localization patterns of the fusion proteins.

## Figures and Tables

**Table 1:** Distribution and classification of NBS-LRR gene family members in representative citrus species and closely related taxa.

	*Citrus reticulata*	*Citrus clementina*	*Citrus sinensis*	*Citrus maxima*	*Citrus trifoliata*	*Citrus hindsii*
NL	328	240	122	44	134	76
TNL	74	71	24	7	10	8
CNL	12	12	9	11	3	9
RNL	3	3	3	4	4	4
Total	417	326	158	66	151	97

**Table 2:** Detailed annotation of the 24 genes in the *C. clementina* genome.

No	Gene	mRNA	Annotation	TM
1	Ciclev10023260m	Detected	LRR/NBS-LRR-ARC,disease resistance	0
2	Ciclev10018540m	Detected	LRR/NBS-LRR-ARC,disease resistance	0
3	Ciclev10023953m	Detected	Mitochondrial ribosomal protein L11	0
4	Ciclev10018510m	Detected	LRR/NBS-LRR-ARC,disease resistance	0
5	Ciclev10024474m	Detected	No annotation	0
6	Ciclev10023481m	Detected	NBS-AFC,partial	0
7	Ciclev10022922m	Detected	No annotation	0
8	Ciclev10019166m	Detected	No annotation	0
9	Ciclev10020079m	Detected	F-box family protein	0
10	Ciclev10023014m	Detected	F-box/RNI-like superfamly protein	0
11	Ciclev10023374m	Detected	Uridine-ribohydrolase 2	0
12	Ciclev10019447m	Detected	Inositol 1,3,4-trisphosphate 5/6-kinase 4	0
13	Ciclev10018897m	Detected	LRR/NBS-LRR-ARC,disease resistance	0
14	Ciclev10019649m	Detected	RNA-binding protein	0
15	Ciclev10021021m	Detected	Chloroplast outer emvelope protcin 37	0
16	Ciclev10024361m	Detected	S-adenosyl.L-methionine methyltransferase	0
17	Ciclev10024293m	Detected	Endonuclease/exonucleaso/phosphatase	0
18	Ciclev10019293m	Detected	Ankyrin repeattamily protein	4
19	Ciclev10023674m	Detected	No annotation	0
20	Ciclev10023198m	Undetected	Pectin lyase-like superfamily protein	0
21	Ciclev10018637m	Detected	LRR receptor-like protein kinase family	1
22	Ciclev10023998m	Undetected	Mitochondrial pynvate carrier 2	0
23	Ciclev10023511m	Undetected	LRR receptor-like protein kinase family	1
24	Ciclev10024127m	Undetected	Chloroplast outer envelope protein 37	0

**Table 3:** Molecular docking results: Vina scores of NBS-LRR2 proteins from *C. reticulata* and *C. clementina* with ACT-I and six additional host-selective toxins.

	ACT-I	ACT-II	ACR-I	AK-I	AF-I	AF-II	AM-I
CrLRR2	−8.2	−7.6	−8.3	−8.1	−6.9	−7.0	−9.3
CcLRR2	−7.5	−8.0	−7.8	−7.4	−6.6	−7.2	−9.5

## Data Availability

The genome-wide identification of NBS-LRR genes was performed using publicly available reference genomes. The *Citrus clementina* genome assembly (GCA_000493195.1) was downloaded from the NCBI database (https://www.ncbi.nlm.nih.gov/). The *Citrus reticulata* (Ponkan) genome data were obtained from the CPBD database (http://citrus.hzau.edu.cn/download.php, accessed on 15 September 2025). The sequences of the *LRR2* gene identified in this study are provided in Table S3. All other data generated or analyzed during this study are included in this article and its Supplementary Materials.

## Supplementary Materials

The following supporting information can be downloaded at: https://www.mdpi.com/article/10.3390/plants15081191/s1. Table S1: Characteristics of DNA sequences and encoded protein molecules of NLR family genes in *C. reticulata*; Table S2: Characteristics of DNA sequences and encoded protein molecules of NLR family genes *C. Clementina.* Table S3: The sequence information of LRR2.
